# Substances Secreted by *Lactobacillus* spp. from the Urinary Tract Microbiota Play a Protective Role against *Proteus mirabilis* Infections and Their Complications

**DOI:** 10.3390/ijms25010103

**Published:** 2023-12-20

**Authors:** Dominika Szczerbiec, Mirosława Słaba, Agnieszka Torzewska

**Affiliations:** 1 Department of Biology of Bacteria, Faculty of Biology and Environmental Protection, University of Lodz, Banacha 12/16, 90-237 Lodz, Poland; dominika.szczerbiec@biol.uni.lodz.pl; 2 Department of Industrial Microbiology and Biotechnology, Faculty of Biology and Environmental Protection, University of Lodz, Banacha 12/16, 90-237 Lodz, Poland; miroslawa.slaba@biol.uni.lodz.pl

**Keywords:** *Proteus mirabilis*, *Lactobacillus*, organic acid, urinary tract infections, urolithiasis

## Abstract

*Proteus mirabilis* urinary tract infections can lead to serious complications such as development of urinary stones. *Lactobacillus* spp., belonging to the natural microbiota of the urinary tract, exhibit a number of antagonistic mechanisms against uropathogens, including the secretion of organic acids. In this study, we determined the anti-adhesion, anti-cytotoxicity and anti-crystallization properties of the substances secreted by *Lactobacillus*. For this purpose, membrane inserts with a pore diameter 0.4 μm were used, which prevent mixing of cultured cells, simultaneously enabling the diffusion of metabolic products. The intensity of crystallization was assessed by measuring the levels of Ca^2+^, Mg^2+^ and NH_3_ and by observing crystals using microscopic methods. The cytotoxicity of the HCV-29 cell line was determined using the LDH and MTT assays, and the impact of lactobacilli on *P. mirabilis* adhesion to the bladder epithelium was assessed by establishing CFU/mL after cell lysis. It was shown that in the presence of *L. gasseri* the adhesion of *P. mirabilis* and the cytotoxicity of the cells decreased. The degree of crystallization was also inhibited in all experimental models. Moreover, it was demonstrated that *L. gasseri* is characterized by the secretion of a high concentration of L-lactic acid. These results indicate that L-lactic acid secreted by *L. gasseri* has a significant impact on the crystallization process and pathogenicity of *P. mirabilis*.

## 1. Introduction

*Proteus mirabilis* is a Gram-negative bacterium, widely distributed in nature, including the human intestine, where it is a component of natural microbiota. It is also a common pathogen of the gastrointestinal and urinary tracts. Urinary tract infections caused by *P. mirabilis* are most often associated with urinary tract catheterization (CAUTIs) [1,2]. *P. mirabilis* accounts for 1–2% of all urinary tract infections (UTIs) and 20–45% of complicated UTIs, e.g., infections associated with long term catheterization, worldwide [3]. Urinary tract infections caused by the bacteria of the genus *Proteus* begin with the colonization of the urinary tract and migration of the pathogen to the bladder and kidneys. These stages are made possible by numerous virulence factors, including the presence of fimbriae, which enable the adhesion of bacteria to the urinary tract epithelium, swarming motility, which is crucial in dissemination of bacteria and production of urease-metaloenzyme, which catalyzes the hydrolysis of urea to ammonia and carbon dioxide. Ammonia released by the action of urease has a toxic effect, damaging the epithelial cells of the urinary tract and promoting infections [4,5]. *P. mirabilis* infections can lead to numerous complications such as pyelonephritis, bacteriuria and development of infection-related urinary stones (also called infectious stones or infection stones). The formation process of these stones is related to the action of urease and they constitute up to 15% of all kidney stones [6]. The mechanism of their formation begins with an increase in urine pH due to the urease activity. Urea hydrolysis into ammonia and carbon dioxide causes an increase in the concentrations of NH_4_^+^, PO_4_^3−^ and CO_3_^2−^ ions. Cooperatively with Mg^2+^ and Ca^2+^ ions, it leads to the formation of struvite (MgNH_4_PO_4_·6H_2_O) and carbonate apatite (Ca_10_(PO_4_)_6_CO_3_) [7]. This process is one of the first stages of urinary stone development and consists of the precipitation of mineral components of urine caused by their excessive concentration in relations to the solubility which leads to crystallization. Struvite and apatite crystals may be washed out by the urine stream or crystals retention may occur in the urinary system. It leads to aggregation and rapid formation of larger forms in the lumen of the bladder or within the kidneys. As a result, it can lead to dangerous complications such as sepsis, renal failure or blocking of the flow of urine through catheters [6].

The structure of urinary stones and the presence of deep crevices allow bacteria to survive during antibiotic therapy and protect them from host immune defense. Moreover, *P. mirabilis* bacteria are able to invade cells and persist inside them, which can lead to intracellular crystallization [8]. Those facts have a huge impact on the treatment of infectious urolithiasis, which is a disease characterized by frequent relapses, ineffective treatment and lack of preventive measures without side effects. The treatment of urease-induced stones includes antibiotic therapy to eliminate the source of infection and removal of the stone using percutaneous nephrolithotomy (PCNL) or shock wave lithotripsy (SWL). Additionally, pharmacologic preventative measures including acidification of the urine or inhibition of urease are applied [6,9,10].

Due to the difficulties in treatment, new therapy options are being sought. It may be interesting to use microorganisms that naturally inhabit the urinary tract. The microbiota of the urinary tracts ensures homeostasis and is crucial in the maintenance of health. The microbes interact with pathogens in multiple ways by producing antimicrobial substances, competing with pathogens for common resources or enhancing epithelial defenses including immune defenses [11]. *L. jensenii* and *L. gasseri* are among the *Lactobacillus* strains most frequently isolated from the urinary tract [12]. The principle extracellular molecules secreted by lactobacilli are organic acids. There are many reports about organic acids and their inhibitory activity against various urogenital tract pathogens such as *Candida albicans*, *C. glabrata* [13], *Chlamydia trachomatis* [14] and *Escherichia coli* [15]. So far, many works have focused on the impact of probiotic strains on the health of the vaginal system; however, there are few reports on the influence of lactobacilli, which belong to the natural microbiota of the urinary tract, and the substances secreted by them on UTIs and infectious stones development.

The purpose of the present study was to investigate the influence of extracellular substances secreted by *L. jensenii* and *L. gasseri* on the adhesion of *P. mirabilis* to the bladder epithelium and cell cytotoxicity. Moreover, the research was aimed at determining the intensity of crystallization caused by *P. mirabilis* in the presence of these substances.

## 2. Results

### 2.1. Extracellular Substances Secreted by Lactobacillus May Reduce the Adhesion of P. mirabilis Strains to the Bladder Epithelium In Vitro

**Figure 1:**
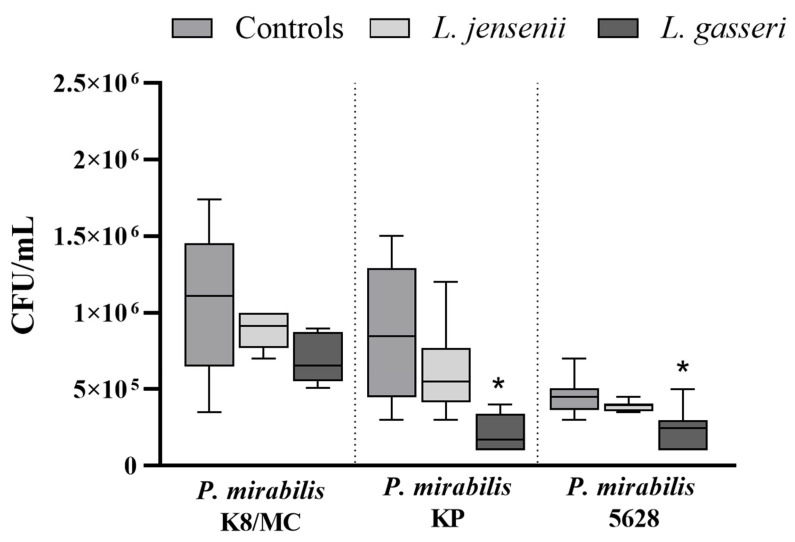
Adhesion of *P. mirabilis* to the bladder epithelium cell line HCV-29 during the incubation in the presence of substances secreted by *Lactobacillus* using the cell culture inserts system. The results are presented as median ± interquartile range (IQR) of three experiments. * *p* < 0.05 for comparison of the number of *P. mirabilis* bacteria (CFU/mL) in pure culture vs. in the presence of extracellular substances of *Lactobacillus*, Kruskal–Wallis test with Dunn’s multiple comparisons.

One of the first steps in *P. mirabilis* urinary tract infections is the adhesion of bacteria to the urothelium surface. This work was focused on the impact of the substances secreted by *Lactobacillus* on this process. The results of anti-adhesion properties of *L. jensenii* and *L. gasseri* against *P. mirabilis* strains are shown in Figure 1. The adhesion of *P. mirabilis* KP and 5628 was significantly reduced in the presence of the substances secreted by *L. gasseri* strain, whereas such a phenomenon was not observed in the presence of *L. jensenii*. Extracellular substances of *L. gasseri* reduced the adhesion of the *P. mirabilis* KP strain to the urothelium by up to 70% (* *p* < 0.05) and in the case of *P. mirabilis* 5628 the reduction was up to 45% (* *p* < 0.05). There were no significant changes observed in the adhesion of *P. mirabilis* K8/MC to the epithelium in the presence of the substances secreted by *Lactobacillus* strains.

**Figure 2:**
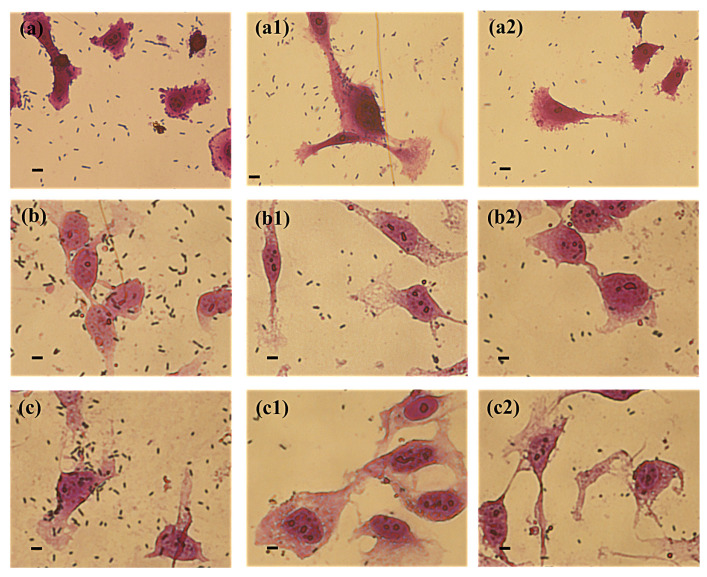
Adhesion of the *P. mirabilis* strains ((**a**) K8/MC; (**b**) KP, (**c**) 5628) to the bladder epithelium cell line HCV-29 in the presence of extracellular substances secreted by *L. jensenii* (**1**) and *L. gasseri* (**2**) using the cell culture inserts system. The scale bar represents 10 μm.

The inhibition of the adhesion of *P. mirabilis* to the bladder epithelium as a result of the action of the extracellular substances of *Lactobacillus* was also confirmed by microscopic observation (Figure 2). All *P. mirabilis* strains showed the ability to adhere to the HCV-29 cell line in vitro as well as to the surface of the polystyrene plate. However, in the presence of the substances secreted by the *L. gasseri* strain, an inhibition in the number of the *P. mirabilis* KP and 5628 bacteria and their adherence properties was observed, which was consistent with the obtained quantitative results.

### 2.2. The Impact of the Substances Secreted by Lactobacillus on Cell Cytotoxicity Induced by P. mirabilis

**Figure 3:**
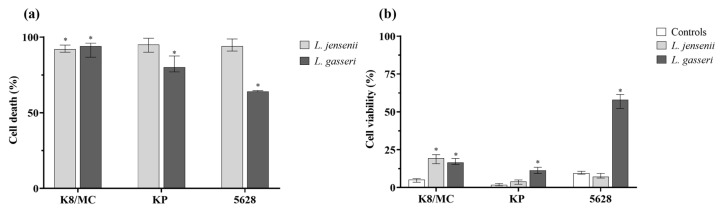
Percentage of cell death in the presence of the extracellular substances secreted by *L. jensenii* and *L. gasseri* strains, where 100% was pure *P. mirabilis* and bladder cell line HCV-29 in RPMI with synthetic urine (**a**). Percentage of cell viability in pure culture of *P. mirabilis* strains and bladder cell line HCV-29 in RPMI with synthetic urine as controls and in the presence of the extracellular substances secreted by *L. jensenii* and *L. gasseri* strains (**b**). The results are presented as median ± interquartile range (IQR) of four experiments. * *p* < 0.05 for the comparison of cell death and cell viability of *P. mirabilis* bacteria in the presence of *L. jensenii* and *L. gasseri* strains vs. in pure cultures. Mann–Whitney U test.

The LDH and MTT assays were performed to investigate the role of the extracellular substances on the cytotoxicity effect induced by the *P. mirabilis* strains on the bladder epithelium. The LDH assay is based on the measurement of the levels of lactate dehydrogenase released into the culture medium upon plasma membrane damage and is expressed as a percentage of cell death, whereas the MTT assay is used to measure cellular metabolic activity and can be expressed as a percentage of cell viability [16]. The level of bladder cell cytotoxicity induced by the *P. mirabilis* strains after 24 h was high. The percentage of eukaryotic cell death caused by all *P. mirabilis* strains in the presence of *L. jensenii* was also high (on average 90%, where 100% were *P. mirabilis* pure cultures) (Figure 3a). However, *L. gasseri* significantly reduced this effect in the co-culture with all *P. mirabilis* strains (* *p* < 0.05). The strongest inhibition of eukaryotic cell death (by 35%) was noticed in the culture with *P. mirabilis* 5628 and extracellular substances of *L. gasseri*. Conversely, as shown in Figure 3b, cell viability after 24 h of incubation was low and remained at the level of 3% for *P. mirabilis* KP and 10% for *P. mirabilis* K8/MC. The viability of eukaryotic cells in the samples with the addition of *Lactobacillus* strains was higher than in those with *P. mirabilis* alone. In particular, the substances secreted by *L. gasseri* had an impact on cell cytotoxicity induced by *P. mirabilis* 5628, where even five times higher cell viability was noticed (* *p* < 0.05).

### 2.3. Extracellular Substances of Lactobacillus Inhibit the Crystallization of Urine Components Caused by P. mirabilis

**Figure 4:**
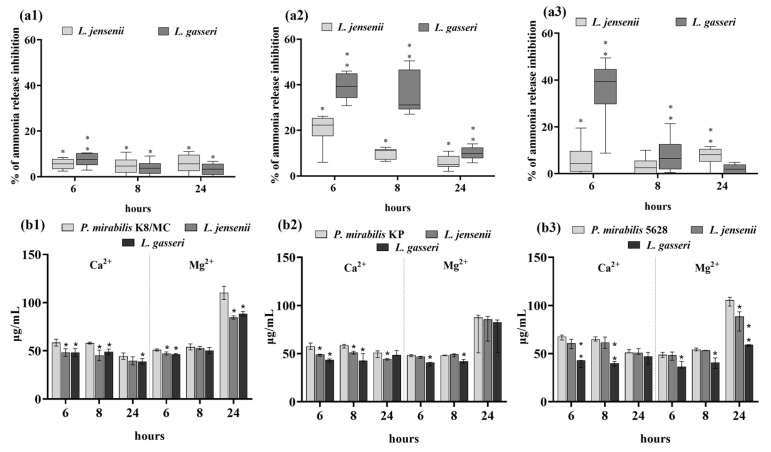
Percentage of ammonia release inhibition in the culture with the *P. mirabilis* strains in synthetic urine and the extracellular substances secreted by *Lactobacillus* after 6, 8 and 24 h of incubation (**a**). Intensity of crystallization expressed as the concentration of Mg^2+^ and Ca^2+^ in the culture with *P. mirabilis* strains in synthetic urine and the extracellular substances secreted by *Lactobacillus* after 6, 8 and 24 h of incubation (**b**). (**1**) corresponds to *P. mirabilis* K8/MC, (**2**) to *P. mirabilis* KP and (**3**) to *P. mirabilis* 5628. The results are presented as median ± interquartile range (IQR) of three experiments. ** *p* < 0.03, * *p* < 0.05 for comparison of ammonia release in pure culture vs. in the presence of *Lactobacillus* extracellular substances using Kruskal–Wallis test with Dunn’s multiple comparisons and for comparison of Ca^2+^ and Mg^2+^ ion concentrations in pure culture vs. in the presence of *Lactobacillus* extracellular substances using Mann–Whitney U test.

The influence of the *Lactobacillus* strains and the secreted substances on the intensity of crystallization was assessed by determining the degree of ammonia release and concentrations of Mg^2+^ and Ca^2+^ ions and by observing the crystals under a microscope. Urease hydrolyzes urea, which leads to an increase in the pH value and release of ammonia. As shown in Figure 4, in the presence of *L. gasseri* and *L. jensenii*, ammonia release was inhibited compared to the controls—pure *P. mirabilis* cultures in synthetic urine. Especially the *L. gasseri* strain showed such properties in the co-culture with *P. mirabilis* KP, where after 6 h of incubation the ammonia release inhibition reached the level of 40% (** *p* < 0.03) compared to the control. In the culture with *P. mirabilis* K8/MC, the *Lactobacillus* strains showed the weakest ability to inhibit the release of ammonia, which remained at a maximum of 10% at 6 h and then decreased till 24 h of the experiment.

In the presence of *L. gasseri*, lower concentrations of Ca^2+^ and Mg^2+^ ions compared to the controls were also observed. This trend was the strongest in the culture with *P. mirabilis* 5628, where the calcium content after 6 h of incubation was almost two times lower than in control (** *p* < 0.03). In the culture of *P. mirabilis* K8/MC and *P. mirabilis* KP, a decrease in the calcium content was noted in the presence of both *Lactobacillus* strains mainly after 6 and 8 h of incubation. Also, only a slight decrease in the magnesium content was observed in the presence of *L. gasseri* after 6 and 8 h of incubation with all *P. mirabilis* strains. After 24 h of incubation, there was a decrease in Mg^2+^ concentration in the culture with *P. mirabilis* K8/MC and 5628 and substances secreted by *Lactobacillus*.

**Figure 5:**
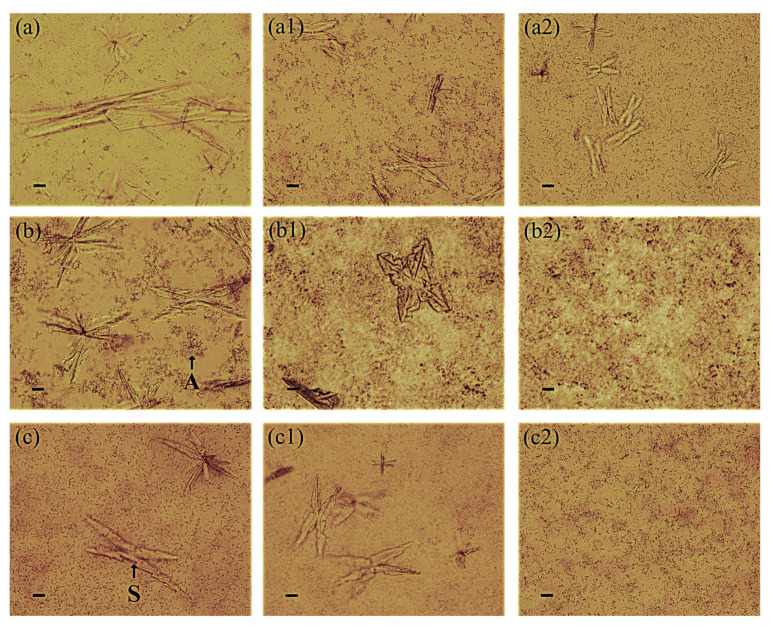
Crystallization caused by *P. mirabilis* ((**a**) *P. mirabilis* K8/MC; (**b**) *P. mirabilis* KP; (**c**) *P. mirabilis* 5628) in the presence of the extracellular substances secreted by *L. jensenii* (**1**) and *L. gasseri* (**2**) after 8 h of incubation. The scale bar represents 10 μm. A corresponds to carbonate apatite and S to struvite crystals.

Phase-contrast microscopy images confirmed that in the samples with *P. mirabilis* 5628 and *P. mirabilis* KP and in the presence of the *L. gasseri* strain, the crystallization of urine components was inhibited (Figure 5). After 8 h of the experiment, no struvite crystals, with an X-shaped dendrite appearance, were observed in the cultures of *P. mirabilis* KP and 5628 in the presence of extracellular substances secreted by *L. gasseri*. Additionally, in the sample with *P. mirabilis* K8/MC and in the presence of *L. jensenii* and *L. gasseri*, the size of struvite crystals was smaller compared to that found in the controls. After 6 h of incubation there were no struvite crystals observed, and after 24 h, struvite and apatite crystals were detected in each sample, therefore the attention was focused on images after 8 h of incubation. Apatite was formed in all samples probably because it crystallizes at a lower pH than struvite.

**Figure 6:**
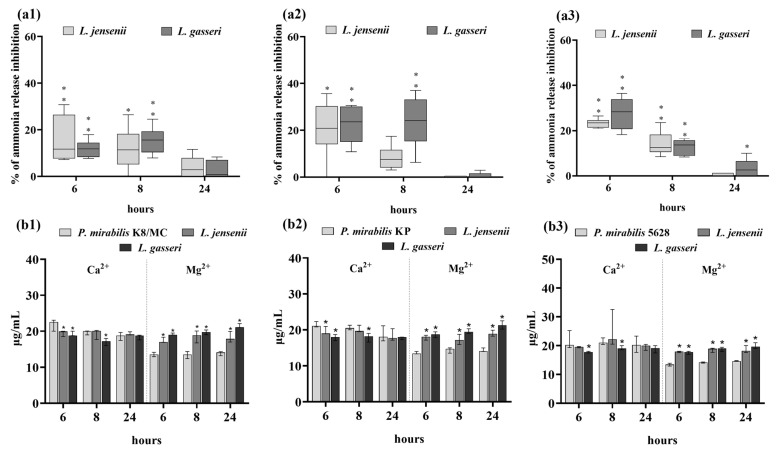
Percentage of ammonia release inhibition in the culture with the *P. mirabilis* strains and the HCV-29 cell line in RPMI medium with synthetic urine and the extracellular substances secreted by *Lactobacillus* after 6, 8 and 24 h of incubation (**a**). Intensity of crystallization expressed as the concentration of Mg^2+^ and Ca^2+^ in the culture with the *P. mirabilis* strains and the HCV-29 cell line in RPMI medium with synthetic urine and the extracellular substances secreted by *Lactobacillus* after 6, 8 and 24 h of incubation (**b**). (**1**) corresponds to *P. mirabilis* K8/MC, (**2**) to *P. mirabilis* KP and (**3**) to *P. mirabilis* 5628. The results are presented as median ± interquartile range (IQR) of three experiments. ** *p* < 0.03, * *p* < 0.05 for comparison of ammonia release in pure culture vs. in the presence of *Lactobacillus* extracellular substances using Kruskal–Wallis test with Dunn’s multiple comparisons, and for comparison of Ca^2+^ and Mg^2+^ ions concentrations in pure culture vs. in the presence of *Lactobacillus* extracellular substances using Mann–Whitney U test.

The intensity of crystallization associated with the epithelium was determined on the basis of the concentration of calcium and magnesium ions, release of ammonia and visualization of crystallization using Von Kossa staining and confocal microscopy. As shown in Figure 6, in the presence of *L. gasseri*, the content of Ca^2+^ ions was significantly lower compared to the controls after 6 or 8 h of incubation. However, the concentration of magnesium in the samples incubated with *Lactobacillus* strains was unexpectedly higher compared to the controls (by up to 20%, * *p* < 0.05). Both *Lactobacillus* strains inhibited ammonia release in the co-culture with all *Proteus* strains. The strongest inhibition of ammonia release (by 27%) was observed in the presence of *L. gasseri* and *P. mirabilis* 5628 after 6 h of incubation (** *p* < 0.03).

**Figure 7:**
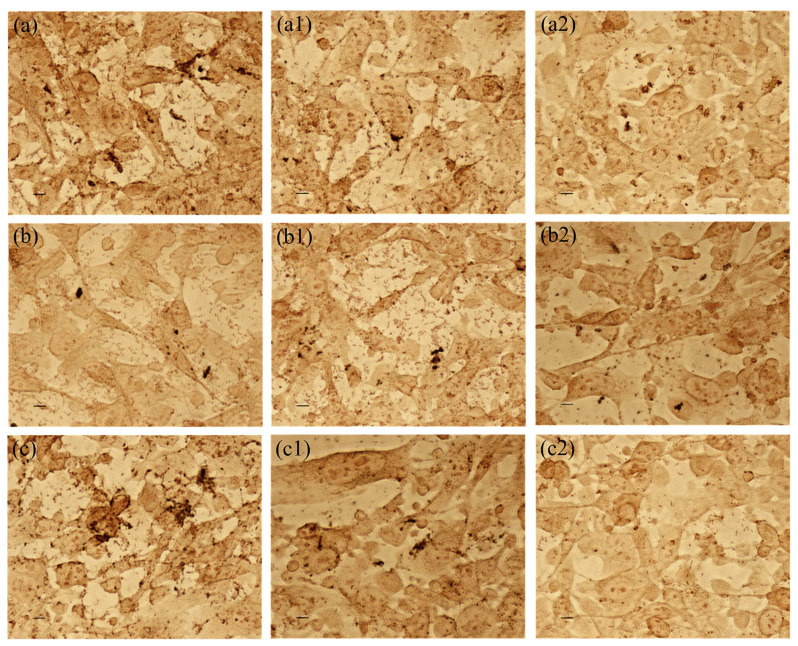
Crystallization caused by *P. mirabilis* ((**a**) *P. mirabilis* K8/MC; (**b**) *P. mirabilis* KP; (**c**) *P. mirabilis* 5628) in the presence of extracellular substances secreted by *L. jensenii* (**1**) and *L. gasseri* (**2**). The scale bar represents 10 μm.

Von Kossa staining was performed to visualize calcium deposits associated with the HCV-29 cells. Calcium is the main component of carbonate-apatite crystals and, as shown in Figure 7, apatite crystals may be formed and remain in the epithelial cells of the bladder and outside the cells. Calcium deposits (black points) inside and outside the cells were observed in all control samples and in some samples with the *Lactobacillus* strains, which proved the ongoing crystallization process. However, no deposits were observed in the presence of the metabolic products of the *L. gasseri* strain and *P. mirabilis* 5628.

**Figure 8:**
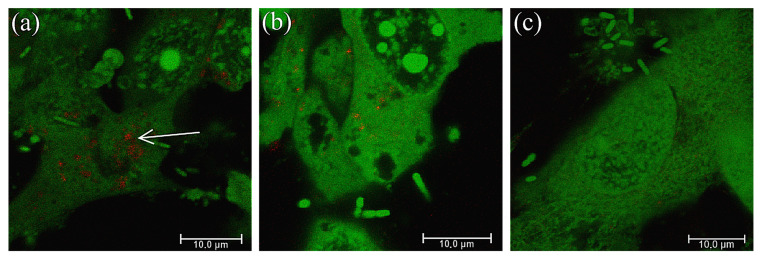
Confocal laser scanning microscopy images. The HCV-29 cell line and the *P. mirabilis* 5628 cells were stained with SYTO-13 and are green, while the crystals show autofluorescence and are red (white arrow). (**a**) corresponds to control, (**b**) corresponds to extracellular substances of *L. jensenii* and (**c**) to *L. gasseri*. The scale bar represents 10 μm.

Images from confocal microscopy allowed for visualization of eukaryotic and bacterial cells, stained by a green fluorescent dye, and crystals showing autofluorescence. The impact of the substances secreted by *Lactobacillus* on crystallization caused by the *P. mirabilis* strains was investigated; however, in confocal microscopy, the attention was focused on *P. mirabilis* 5628 in a co-culture with *L. gasseri,* where the intensity of crystallization was inhibited the most intensively. As shown in Figure 8, autofluorescent crystals (red points) were most noticeable in the control sample with *P. mirabilis* 5628, inside the eukaryotic cells (Figure 8a). There were no reflecting crystals in the presence of the substances secreted by *L. jensenii* (Figure 8b) and *L. gasseri* (Figure 8c). Additionally, it could be observed that in the presence of the *Lactobacillus* extracellular substances, especially substances of *L. gasseri*, eukaryotic cells looked healthier compared to control, which proves the inhibition of the cytotoxic effect of *Proteus*.

**Figure 9:**
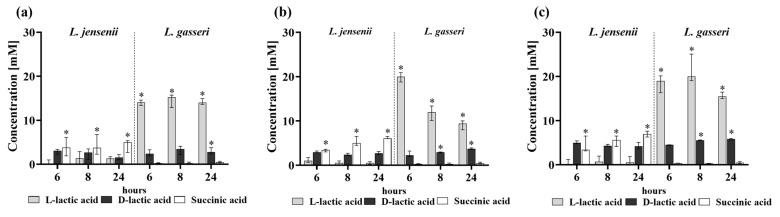
Concentrations [mM] of L-lactic, D-lactic and succinic acids after 6, 8 and 24 h of incubation with *P. mirabilis* strains ((**a**) K8/MC, (**b**) KP, (**c**) 5628) and in the presence of *L. jensenii* and *L. gasseri* using the cell culture inserts system. The results are presented as median ± interquartile range (IQR) of four experiments. * *p* < 0.05 for the comparison of concentration of organic acids in the tested supernatants in the presence of *L. jensenii* vs. in the presence of *L. gasseri* according to Mann–Whitney U test.

In our previous studies, we had shown that organic acids secreted by the tested *Lactobacillus* strains had antibacterial properties [17] and L-lactic acid was able to inhibit the activity of urease, an enzyme secreted by *Proteus* strains involved in the crystallization process [18]. The two main organic acids secreted by the tested strains of *L. gasseri* and *L. jensenii* are lactic acid and succinic acid; therefore, in this work the attention was focused on the secretion of these two acids. The results of this study indicate that *L. gasseri* and their extracellular substances can inhibit the crystallization process and the cytotoxicity of bladder cells induced by *P. mirabilis* and have the ability to inhibit the adhesion of these uropathogens to the epithelium. Simultaneously, this strain produces a higher concentration of L-lactic acid compared to *L. jensenii* (Figure 9). Interestingly, *L. jensenii* may be distinguished by the secretion of a high concentration of succinic acid, and both strains produce similar level of D-lactic acid. These results indicate that L-lactic acid may be responsible for the anti-adhesive, anti-crystallization or anti-cytotoxic properties.

## 3. Discussion

Infectious urolithiasis is one of the subsets of urolithiasis characterized by a high rate of morbidity and mortality, constituting a global health-care issue. Struvite stones are formed rapidly within 4–6 weeks, treatment of this disease is often ineffective and relapses are very common [19,20]. According to the research of Wong. et al. [21], after a complete eradication of a urinary stone, recurrences were observed at the level of 10%; however, if fragments of the stone remained in the urinary tract, the recurrence rate increased to 85%. Moreover, difficulties in the treatment can be also associated with the fact that bacteria can hide in the interstices of the stone or exist within eukaryotic cells, which reduces the effectiveness of antibiotic therapy [8,22]. All these facts make infectious urinary stones the most challenging to treat among all types of stones.

Urease-producing microorganisms, bacteria belonging to the genus *Proteus* in particular, are involved in the formation of urinary stones. Bichler et. al. [23] demonstrated that 100% of *Proteus* strains produce this enzyme and studies conducted by Kramer et al. [24] showed that 70% of urinary stone isolates were microorganisms belonging to this genus. In our previous studies, we had demonstrated that organic acids of the tested *Lactobacillus* strains had antibacterial properties against *P. mirabilis* [17] and lactic acid had an impact on urease activity [18]. Additionally, we showed that *Lactobacillus* inhibited crystallization in the co-culture with *P. mirabilis*. This knowledge led us to speculate that organic acids secreted by *Lactobacillus* affected the crystallization process as well as the adhesion of the *Proteus* strains to the bladder epithelium in vitro and decreased cell cytotoxicity induced by these uropathogens.

The first stage of UTIs and development of urease-induced stones is the invasion of uropathogens and their adhesion to the epithelium. *P. mirabilis* uses many virulence factors to initiate the infection [25]. Ammonia produced by *P. mirabilis* also has a great impact on the adhesion of bacteria to the urothelium and its colonization. Ammonia damages the layers of glycosaminoglycan that cover the epithelium of the urinary tract [20]. Struvite and apatite crystals can be formed in the bladder as well as in the kidneys. Their aggregation leads to the formation of urinary stones, which may damage the epithelium. At the same time, these areas can serve as nucleation sites for the crystallization process [26]. The presented results indicate that the *L. gasseri* strain reduces the adhesion of the *P. mirabilis* strains to the bladder epithelium by secreting extracellular substances. There are many reports about the anti-adhesion properties among *Lactobacillus* species concerning their inhibitory effect on the adhesion of pathogens to the genital tract [27] or urinary tract [28]. There are several mechanisms that allow *Lactobacillus* to protect the epithelium against pathogens. Lactic acid bacteria secrete extracellular substances such as organic acids or bacteriocins, which have an antibacterial effect, secrete proteins that degrade carbohydrate receptors and have an ability to produce receptor analogs and biosurfactants. The inhibition of adhesion can occur by displacing pathogens from the epithelium cells and by competition with the pathogen for specific receptors [29].

In the course of experiments, the influence of the extracellular substances secreted by the tested *Lactobacillus* strains on the cytotoxic effect induced by *P. mirabilis* strains was investigated. As shown in this study, *L. gasseri* significantly inhibited eukaryotic cell death caused by all *P. mirabilis* strains tested. Similarly, the viability of the cells after 24 h of incubation was the highest in the presence of *L. gasseri* extracellular components. We suggest that these anti-adhesion and anti-cytotoxicity properties of the *L. gasseri* strain are the result of L-lactic acid action, due to that fact that it is the main organic acid produced by this strain with antibacterial and urease-inhibitor activity. Maudsdottere et al. [30] showed that lactic acid produced by *Lactobacillus* exhibited antibacterial properties against *Streptococcus pyogenes* and reduced cytotoxicity of pharyngeal epithelial cells. Lactic acid had an ability to degrade *S. pyogenes* lipoteichoic acid, which induced host cell cytotoxicity. Therefore, it can be assumed that, in addition to the mechanisms indicated by our work, this process may be also influenced by others, operating simultaneously.

In our previous paper, we had revealed that *Lactobacillus* might inhibit the crystallization process caused by *P. mirabilis* with different intensity [18]. In this work, we focused on two *Lactobacillus* strains that showed the strongest and the weakest ability of crystallization inhibition. Additionally, to confirm the fact that extracellular components are responsible for those properties, a system with inserts was used, which allowed diffusion of *Lactobacillus* metabolic products without mixing the *Proteus* and *Lactobacillus* cells. In the current research, we observed that the substances produced by *L. gasseri* significantly reduced the release of ammonia, and lower concentrations of Mg^2+^ and Ca^2+^ ions indicated the inhibition of the crystallization process. *L. jensenii* also exhibited such properties but to a lesser extent. The characteristic appearance of X-shaped struvite crystals formed due to the rapid increase in the pH value was also confirmed by phase contrast microscopy [31]. Similar results were obtained in the assay of crystallization associated with the epithelium. *Lactobacillus* strains, especially *L. gasseri*, inhibited the release of ammonia and calcium concentration. However, the Mg^2+^ ions concentration was different from that observed during the crystallization without the epithelium. In the presence of *Lactobacillus* strains, the content of Mg^2+^ ions were higher compared to the control. However, this was not due to the increased intensity of struvite crystallization but to the presence of a larger number of epithelial cells compared to the control system. A higher cell number was associated with reduced cytotoxicity of the bacteria. The estimated level of intracellular magnesium is 15–20 mM [32], while the level of calcium is approximately 0.1–0.5 μM [33]; hence, in the experiments in the presence of the epithelium, the level of cellular magnesium but not calcium might have interfered with the assessment of intensity of crystallization. Microscopic observations confirmed the inhibitory effect of the substances secreted by *Lactobacillus* on the crystallization induced by *P. mirabilis*. The reduced intracellular calcium content in the presence of *L. gasseri* was confirmed using Von Kossa staining and confocal microscopy images. There is not much data concerning the autofluorescence of urinary crystals. However, Mathoera et al. [34] visualized this phenomenon as well as intracellular crystallization in the ureter cell line SV-HUC-1. Also, Lin et al. [35] proved that calcium oxalate dehydrates, uric acid and calcium oxalate monohydrate crystals showed autofluorescence.

The *Lactobacillus* strains differ in their antibacterial properties, as shown by the research conducted by Hutt et al. [36], where *L. crispatus* were found to have a stronger antagonistic effect against *E. coli* and *Candida* than *L. jensenii* and *L. gasseri*. The concentration and type of secreted organic acids may also depend on the species of the microorganisms, culture medium and conditions [37]. Our study revealed that the tested *Lactobacillus* strains differed in terms of the production of organic acids in synthetic urine environment. *L. jensenii* was found to secrete large amounts of succinic acid, while *L. gasseri* produced L-lactic acid. Lactic acid exists in two enantiomers; L-lactic acid is an endogenous compound and D-lactic acid is characterized by harmful properties. D-lactic acid is a toxic metabolite, which in large doses may cause various health complications and has neurotoxic effects on organisms. Under normal conditions, concentration of this acid in the human body is controlled. However, this acid may be overproduced by microbiota in short bowel syndrome and after jejunoileal bypass surgery [38]. *Lactobacillus* strains generally produce both D-lactate and L-lactate, which was confirmed by our research as well. However, the level of concentrations may be different among *Lactobacillus* species, like in the study conducted by Witkin et al. [39] where the level of D-lactic acid in the samples containing *L. crispatus* was higher than in those with *L. iners*.

## 4. Materials and Methods

### 4.1. Bacterial Strains

The *P. mirabilis* strains (KP; 5628) were isolated at the Department of Microbiology from the urine of patients of the Children’s Memorial Health Institute in Warsaw, Poland, who had been diagnosed with infectious urolithiasis. The *P. mirabilis* K8/MC strain was obtained from urinary stones and provided by the Provincial Specialist Hospital M. Pirogow in Lodz. The strains were identified using the API 20E test (Biomerieux, Marcy-I’Etoile, France) and cultured on TSB (tryptic soy broth, BTL, Warsaw, Poland) at 37 °C for 24 h.

*Lactobacillus* strains were isolated from the urinary tract of healthy people with the consent of the Committee for Bioethics of Scientific Research of the University of Lodz (4(I)/KBBN-UŁ/II/2020), and were deposited in the bacterial strain collection at the Department of Biology of Bacteria, University of Lodz. The method of isolation of these strains and their characteristics had been described previously [17]. Briefly, the strains were obtained from human urine of both men and women who had not been treated with antibiotics and probiotics in the last 3 months. They were identified by their morphology and mass spectrometry using a MALDI/TOF Microflex LT (Bruke, Billerica, MA, USA). *Lactobacillus* spp. were cultured on APT broth (BD Difco, Franklin Lakes, NJ, USA) and incubated in 5% CO_2_ at 37 °C for 48 h.

### 4.2. Adhesion Assay

The effect of extracellular substances secreted by *Lactobacillus* strains on the adhesion of the tested *P. mirabilis* strains to the bladder epithelial cell line (HCV-29) was determined in this assay. The cells were grown in RPMI 1640 medium (Biowest, Nuaille, France) supplemented with 10% heat-inactivated fetal calf serum (FBS, Biowest, Nuaille, France), 2 mM ultraglutamine (Lonza, Walkersville, MS, USA), 100 U/mL penicillin and 100 μg/mL streptomycin (PolfaTarchomin, Warsaw, Poland). The assay was carried out on the adherent 12-well plates with polycarbonate membrane inserts with a pore diameter 0.4 μm (Thermofisher, Waltham, MA, USA), seeded with 1 × 10^5^ cells per well. After 5 days of incubation in a humidified incubator with 5% CO_2_ at 37 °C, RPMI medium without antibiotics and synthetic urine (in the ratio 4:1) were added. Synthetic urine contained the following components [g/L]: CaCl_2_·2H_2_O, 0.651; MgCl_2_·6H_2_O, 0.651; NaCl, 4.6; Na_2_SO_4_, 2.3; sodium citrate, 0.65; sodium oxalate, 0.02; KH_2_PO_4_, 2.8; KCl, 1.6; NH_4_Cl, 1.0; urea, 25.0; creatinine, 1.1; tryptic soy broth, 10.0 (Sigma, St. Louis, MO, USA). It was prepared according to the previously described procedure [18,40]. The HCV-29 cell line was infected with bacterial cultures in the ratio 1:5 (it was determined that in this ratio, crystallization was most intensively inhibited by *Lactobacillus*) of *P. mirabilis* and *Lactobacillus*, cultured as described in Section 4.1. The control was pure *P. mirabilis* in the mixture of RPMI and synthetic urine. A bacterial culture of *P. mirabilis* was added to the lower part of the plate and that of *Lactobacillus* to the insert, which ensured the diffusion of metabolic products of *Lactobacillus* while simultaneously preventing the mixing of *Proteus* and *Lactobacillus* cells. After 1 h of incubation, the cells were washed two times with PBS to remove nonadherent bacteria. To establish the number of adherent *P. mirabilis* bacteria, a monolayer was lysed using 1% Triton X-100. Serial dilutions were seeded on Tryptic Soy Agar (TSA, BTL, Warsaw, Poland) and incubated at 37 °C for 24 h and the results were presented as bacterial colony-forming units (CFU/mL). Additionally, in order to assess the adhesion of the *P. mirabilis* strains, the qualitative, crystal violet staining method was performed. In short, the cells were seeded 1 × 10^5^ per well and incubated for 24 h. Subsequently, 4% formaldehyde was added to the wells for 10 s to fix the cells. Then, after washing with PBS solution, 1% crystal violet aqueous solution was added to each well containing epithelial cells with adherent *P. mirabilis* strains and stained for 5 min. After incubation and washing with distilled water, adhesion was observed using a phase-contrast microscope (Nikon Eclipse TE-2000-S, Tokyo, Japan).

### 4.3. In Vitro Cytotoxicity Studies

The influence of *Lactobacillus* extracellular substances on the cytotoxic effect of HCV-29 cells induced by *P. mirabilis* was determined using the MTT and lactate dehydrogenase activity (LDH) assays. The MTT assay was performed according to Toniolo G. et al. [41]. Briefly, HCV-29 cells (1 × 10^5^ cells/per well) were cultured in a 12-well plate for 5 days and infected with *P. mirabilis* and *Lactobacillus* strains as described in Section 4.2. After 24 h of incubation, using the cell culture inserts system, the bacterial cultures were removed and washed two times with PBS solution. Subsequently, 40 μL of MTT solution (5 mg/mL in PBS, Sigma, St. Louis, MO, USA) and 400 μL of RPMI 1640 medium without antibiotics were added to the wells. The cells were incubated under the same conditions for 3 h. The solution was then removed and the formed formazan crystals were dissolved in DMSO and glycine buffer (12.5 mL 0.2 M glycine; 10.9 mL 0.2 M NaOH; 26.6 mL H_2_O). The absorbance was measured on a 96-well plate at 550 nm wavelength using a Multiskan Ex microplate reader (Labsystems, Helsinki, Finland). The results were expressed as a percentage of cell viability, where the positive control was an untreated monolayer of the cell line HCV-29 and the negative control was a monolayer of the cell line infected with the *P. mirabilis* strains.

To measure the level of cell death, the HCV-29 cell line was cultured and infected as described above. The LDH assay was performed using the LDH assay kit (Roche Diagnostics GmbH Roche Applied Science Mannheim, Germany) according to the manufacturer’s protocol. In brief, after 24 h, the culture supernatants were collected and transferred to a 96-well plate. Subsequently, 100 μL of the reaction mixture was added and incubated for 30 min and 50 μL of a stop solution was added to each well. The absorbance was measured at 490 nm and 625 nm wavelengths. The results were expressed as a percentage of cell death, where 100% was the monolayer infected with *P. mirabilis* strains.

### 4.4. Crystallization Assay

The crystallization assay was performed on 12-well plates with polycarbonate membrane inserts with a pore diameter 0.4 μm to assess the influence of extracellular substances of *Lactobacillus* on the crystallization caused by *P. mirabilis* over time. A total of 2 mL of synthetic urine and the cultures of *P. mirabilis* and *Lactobacillus* were added as described in Section 4.2. Pure *P. mirabilis* cultures in synthetic urine were controls in this experiment. The plate was incubated at 37 °C and at 6, 8 and 24 h of incubation the samples were analyzed to evaluate the intensity of crystallization. Ca^2+^ and Mg^2+^ ion concentrations were determined by the atomic absorption spectroscopy method with flame atomization (240FS AA Agilent, Santa Clara, CA, USA). Samples for this analysis were prepared by collecting the supernatants at the tested hours (0.5 mL) and additionally by adding 0.5 mL of 65% HNO_3_ to the emptied wells for mineralization of the residue. Ammonia release in the tested samples was measured by the phenol hypochlorite colorimetric method [42], and the results were expressed as a percentage of the inhibition of ammonia release, where 100% was the concentration of ammonia in the control, synthetic urine with *P. mirabilis.* Qualitative analysis of the degree of crystallization consisted of the observation of struvite and apatite crystals using a phase-contrast microscope (Nikon Eclipse TE-2000-S, Tokyo, Japan). Moreover, at 6, 8 and 24 h, supernatants were collected and the concentrations of L-lactic acid, D-lactic acid and succinic acid were determined, following the manufacturer’s instructions, using D-lactate (MAK058), L-lactate (MAK329) and succinate (MAK335) assays kits (Sigma-Aldrich; St. Louis, MO, USA).

The evaluation of crystallization associated with the epithelium was performed analogously. The HCV-29 cell line was cultured and infected as described in Section 4.2. The plate was incubated at 37 °C and after 6, 8 and 24 h of incubation, the samples were analyzed to assess Ca^2+^ and Mg^2+^ and ammonia release at the same conditions as above.

### 4.5. Imaging of Cell-Associated Crystallization

Von Kossa staining was performed according to Akiyoshi et al. [43] with some modifications to detect and visualize calcium deposits in the tissue samples. The HCV-29 cell line (1 × 10^5^ cells per well) was cultured at 37 °C for 24 h, 5% CO_2_ on the surface of an adherent 12-well plate, and infected with *P. mirabilis* and *Lactobacillus* strains as described in Section 4.2. After 4 h of incubation, the monolayer was washed with distilled water and 5% silver nitrate was added to the wells. After 20 min of exposure to the UV lamp, the wells were washed with distilled water and treated with 5% sodium thiosulphate for 5 min. Subsequently, the monolayer was washed again and stained with 1% neutral red. The red-stained cells with black calcium deposits were observed using a phase contrast microscope (Nikon Eclipse TE2000-S, Tokyo, Japan).

A confocal laser scanning microscope (CLSM) was used to image crystallization using the phenomenon of crystal autofluorescence. The eukaryotic cells were cultured as described above, on an adherent black 12-well plate with a FLux bottom (SPL Life Sciences, Pochon, Republic of Korea) and infected with the *P. mirabilis* and *Lactobacillus* strains as described in Section 4.2. After 4 h of incubation, the monolayer was washed with PBS solution and incubated with 4% of formaldehyde solution for 15 min to fix the cells. Then, after washing the wells twice with PBS, the green fluorescent nucleic acid dye SYTO13 (1:1000 dilution in PBS, Thermofisher, Waltham, MA, USA) was added and the culture was incubated in the dark for 20 min. After incubation, the cells were washed once again with PBS and suspended in 250 μL of PBS solution. The imaging was performed using a Leica TCS SP8 (Wetzlar, Germany), LAS-AF Version 3.3.0 software with a DMI6000 inverted microscope and a 63× magnification and oil immersion objective (HCL APO), HyD, in standard mode. SYTO13 was excited at 488 nm (laser intensity 5%) and 594 nm was used to visualize the autofluorescence of crystals (laser intensity 30%) [35]. Confocal scanning was performed bidirectionally at 400 Hz and the line average was set to 4. Control and testing samples had the same parameters sets.

### 4.6. Statistical Analysis

All experiments were carried out at least in triplicate. The normal distribution was analyzed by the Kolmogorov–Smirnov test and homogeneity of variance was performed by Levene’s test. Statistical analyses were based on the non-parametric Kruskal–Wallis test with Dunn’s multiple comparisons and the Mann–Whitney U test, performed using Statistica software version 13.3 (StatSoft, Krakow, Poland) and GraphPad Prism 9 software (GraphPad Software, San Diego, CA, USA). The results were considered to be statistically significant at *p* value < 0.05.

## 5. Conclusions

On the basis of the above results, it can be concluded that extracellular substances of *Lactobacillus* have an impact on the pathogenicity of *P. mirabilis* strains by inhibiting the adhesion to the epithelium, reducing cytotoxicity and the crystallization process. We indicated that L-lactic acid was responsible for such properties, which seems to confirm our previous research results [18]. Simultaneously, we cannot exclude that other molecules secreted extracellularly by *Lactobacillus*, and other mechanisms that will be the subject of our further work are also responsible for these effects. However, the results of this work may contribute to better treatment and prevention of urinary tract diseases, especially infectious urolithiasis.

## Data Availability

Data are contained within the article.
